# Lamin A/C Ablation Restricted to Vascular Smooth Muscle Cells, Cardiomyocytes, and Cardiac Fibroblasts Causes Cardiac and Vascular Dysfunction

**DOI:** 10.3390/ijms241311172

**Published:** 2023-07-06

**Authors:** Alberto Del Monte-Monge, Íñigo Ruiz-Polo de Lara, Pilar Gonzalo, Carla Espinós-Estévez, María González-Amor, Miguel de la Fuente-Pérez, María J. Andrés-Manzano, Víctor Fanjul, Juan R. Gimeno, Roberto Barriales-Villa, Beatriz Dorado, Vicente Andrés

**Affiliations:** 1 Centro Nacional de Investigaciones Cardiovasculares (CNIC), Melchor Fernández Almagro 3, 28029 Madrid, Spain; alberto.delmonte@cnic.es (A.D.M.-M.); inigo.ruiz@cnic.es (Í.R.-P.d.L.); pgonzalo@cnic.es (P.G.); carla.espinos@cnic.es (C.E.-E.); maria.gonzalez@cnic.es (M.G.-A.); miguel.delafuente@cnic.es (M.d.l.F.-P.); mjandres@cnic.es (M.J.A.-M.); beatrizjulia.dorado@cnic.es (B.D.); 2 Centro de Investigación Biomédica en Red de Enfermedades Cardiovasculares (CIBERCV), 28029 Madrid, Spain; jgimeno@um.es (J.R.G.); rbarrialesv@gmail.com (R.B.-V.); 3 Cardiac Department, Hospital Clínico Universitario Virgen Arrixaca, 30120 Murcia, Spain; 4 Unidad de Cardiopatías Familiares, Complexo Hospitalario Universitario A Coruña (INIBIC-CHUAC), 15006 A Coruña, Spain

**Keywords:** lamin A/C, laminopathies, dilated cardiomyopathy, vascular smooth muscle cell, vascular dysfunction, transgenic mice

## Abstract

Mutations in the *LMNA* gene (encoding lamin A/C proteins) cause several human cardiac diseases, including dilated cardiomyopathies (*LMNA*-DCM). The main clinical risks in *LMNA*-DCM patients are sudden cardiac death and progressive left ventricular ejection fraction deterioration, and therefore most human and animal studies have sought to define the mechanisms through which *LMNA* mutations provoke cardiac alterations, with a particular focus on cardiomyocytes. To investigate if *LMNA* mutations also cause vascular alterations that might contribute to the etiopathogenesis of *LMNA*-DCM, we generated and characterized *Lmna^flox/flox^SM22αCre* mice, which constitutively lack lamin A/C in vascular smooth muscle cells (VSMCs), cardiac fibroblasts, and cardiomyocytes. Like mice with whole body or cardiomyocyte-specific lamin A/C ablation, *Lmna^flox/flox^SM22αCre* mice recapitulated the main hallmarks of human *LMNA*-DCM, including ventricular systolic dysfunction, cardiac conduction defects, cardiac fibrosis, and premature death. These alterations were associated with elevated expression of total and phosphorylated (active) Smad3 and cleaved (active) caspase 3 in the heart. *Lmna^flox/flox^SM22αCre* mice also exhibited perivascular fibrosis in the coronary arteries and a switch of aortic VSMCs from the ‘contractile’ to the ‘synthetic’ phenotype. Ex vivo wire myography in isolated aortic rings revealed impaired maximum contraction capacity and an altered response to vasoconstrictor and vasodilator agents in *Lmna^flox/flox^SM22αCre* mice. To our knowledge, our results provide the first evidence of phenotypic alterations in VSMCs that might contribute significantly to the pathophysiology of some forms of *LMNA*-DCM. Future work addressing the mechanisms underlying vascular defects in *LMNA*-DCM may open new therapeutic avenues for these diseases.

## 1. Introduction

Nuclear A-type lamins are type V filaments that are predominantly located underneath the inner nuclear membrane, where they are important components of the nuclear lamina found in nearly all differentiated mammalian cells [1,2,3]. There are two main A-type lamin proteins, lamins A and C, which are produced through alternative splicing of the same *LMNA* transcript (lamin C spans exons 1–10, while lamin A spans exons 1–12). A-type lamins play a crucial role in maintaining nuclear integrity, structure, and function. They ensure the proper spatial organization and function of chromatin, nuclear pore complexes, and other proteins that interact with nuclear lamins; additionally, A-type lamins are essential for nucleoskeleton–cytoskeleton connections, which are important for signal mechanotransduction to the nucleus [4,5,6]. As a result, lamins A and C regulate various cell functions, including cell proliferation, migration, and differentiation; signal transduction and gene expression; responses to DNA damage; and mechanosensing [4,5,6]. Interestingly, the expression level of A-type lamins correlates with tissue stiffness and the level of mechanical stress that cells experience. They are expressed at low levels in soft tissues such as fat and the brain and at high levels in muscle tissues, where lamins protect the nucleus from high mechanical stress [7,8].

Interest in A-type lamins has increased with the discovery of more than 400 *LMNA* mutations that cause a broad range of human diseases collectively called laminopathies. Laminopathies include systemic progeroid syndromes such as Hutchinson-Gilford progeria syndrome and tissue-specific diseases, such as lipodystrophies, neurological diseases, and a range of disorders affecting skeletal and/or cardiac muscle such as Emery–Dreifuss muscular dystrophy, limb–girdle muscular dystrophy, and dilated cardiomyopathy (DCM) [3,9]. The second most frequent DCM, and the cause of more than 40% of sudden cardiac deaths, is *LMNA*-associated DCM (*LMNA*-DCM), an autosomal dominant genetic disease characterized by cardiac dilation, reduced systolic function, defective atrioventricular conduction, cardiac arrythmias, extensive cardiac fibrosis, and heart failure [10,11,12,13,14,15]. Nearly 20% of *LMNA*-DCM patients require heart transplantation, and sudden cardiac death due to ventricular arrhythmias occurs frequently, often before the DCM becomes symptomatic [16]. There is a lack of specific therapies for *LMNA*-DCM, and patients are currently treated according to the standard heart failure protocol, with those with malignant arrhythmic events receiving an implantable cardioverter defibrillator to prevent sudden cardiac death [15,17]. There is, therefore, an urgent need for preclinical *LMNA*-DCM models that can be used to identify mechanisms that govern disease progression and develop specific therapies that have a real impact on society. Mouse models generated to investigate the molecular and cellular pathogenesis of *LMNA*-DCM include knock-in mice ubiquitously expressing *Lmna* mutations homologous to those that cause the disease in humans (*Lmna^N195K/N195K^* and *Lmna^H222P/H222P^*) and knock-out mice with whole body *Lmna* deficiency, which progressively develop cardiac fibrosis and conduction defects and DCM and die prematurely [18,19,20,21]. Similar to the whole body *Lmna*-null mice, *Lmna^flox/flox^Myh6-Cre* mice with *Lmna* deletion restricted to cardiomyocytes, develop severe cardiac dysfunction and conduction defects, ventricular arrhythmias, cardiac fibrosis, and apoptosis and die within 4 weeks of birth [22,23]. The cardiac phenotype of *LMNA*-DCM is also partially recapitulated in *Lmna^flox/flox^Pdgfra-Cre* mice, in which A-type lamins are absent from ~80% of cardiac fibroblasts and ~25% of cardiomyocytes [24]. Although studies using these cell-type-specific mouse models identified the important role of cardiomyocytes and cardiac fibroblasts in *LMNA*-DCM, they did not address the crosstalk among cardiac and non-cardiac cells, a relevant issue considering that A-type lamins are broadly expressed in mammalian cells. The purpose of this work was to examine the possible involvement in *LMNA*-DCM of *Lmna* deletion in vascular smooth muscle cells (VSMCs), a cell type that plays a critical role in cardiovascular pathophysiology. To this end, we generated *Lmna^flox/flox^SM22αCre* mice to evaluate the effects of combined *Lmna* deficiency in VSMCs, cardiomyocytes, and cardiac fibroblasts, a situation that occurs in *LMNA*-DCM patients and provides a more translational model of the potential cross-talk between these cell types. In addition to developing the expected severe cardiac phenotype and dying prematurely, *Lmna^flox/flox^SM22Cre* mice show VSMC alterations previously unrecognized in the context of *LMNA*-DCM that may play an important role in the etiopathogenesis of this laminopathy.

## 2. Results

### 2.1. Lmna^flox/flox^SM22αCre Mice with Lmna Deficiency Restricted to Vascular Smooth Muscle Cells, Cardiac Fibroblasts, and Cardiomyocytes Die Prematurely

**Figure 1:**
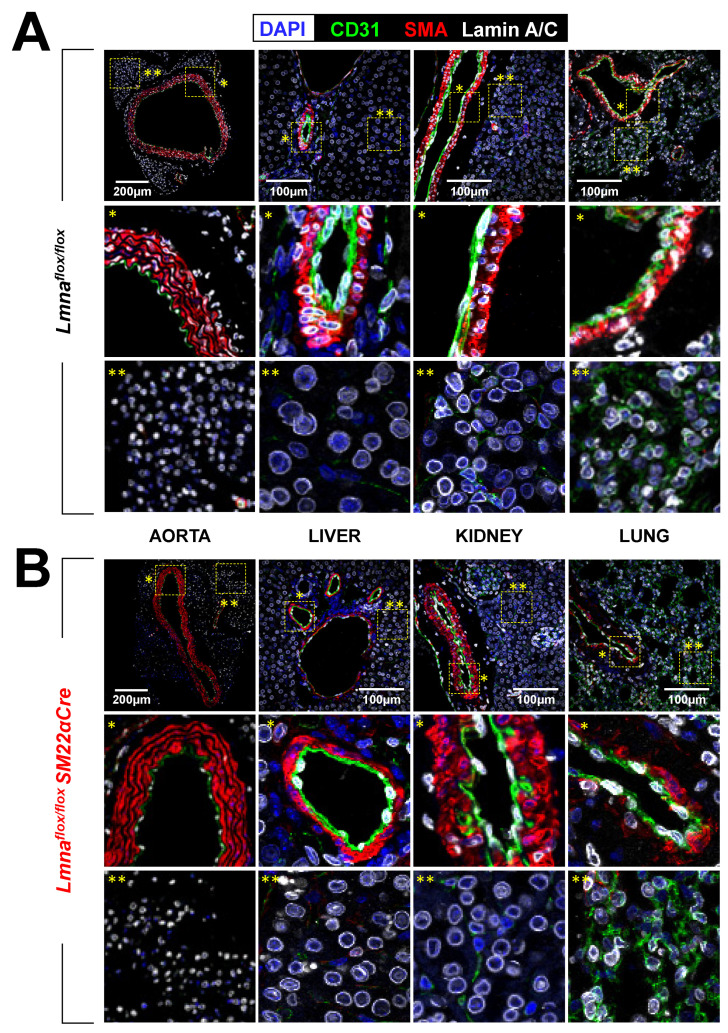
Lamin A/C ablation in VSMCs in *Lmna^flox/flox^SM22αCre* mice. Representative immunofluorescence images of aorta, liver, kidney, and lung from 4-week-old *Lmna^flox/flox^* (**A**) and *Lmna^flox/flox^SM22αCre* mice (**B**). Lamin A/C is visualized in white, endothelial cells in green (anti-CD31 antibody), VSMCs in red (anti-smooth muscle α-actin (SMA) antibody), and nuclei in blue (DAPI staining). Magnified images show vessel-containing regions (*) and vessel-free regions (**).

**Figure 2:**
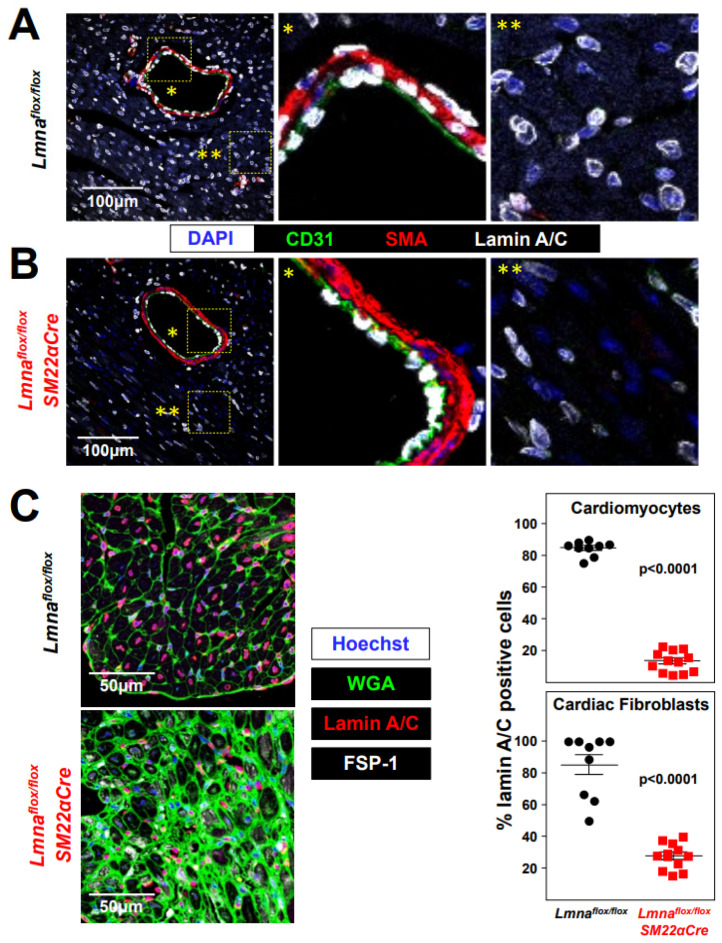
Lamin A/C ablation in heart VSMCs, cardiomyocytes, and cardiac fibroblasts in *Lmna^flox/flox^SM22αCre* mice. (**A**,**B**) Representative immunofluorescence images of heart tissue from 4-week-old *Lmna^flox/flox^* (**A**) and *Lmna^flox/flox^SM22αCre* mice (**B**). Lamin A/C is visualized in white, endothelial cells in green (anti-CD31 antibody), VSMCs in red (anti-smooth muscle α-actin (SMA) antibody), and nuclei in blue (DAPI staining). Magnifications show vessel-containing (*) and vessel-free (**) regions. (**C**) Representative immunofluorescence images of heart tissue from 4-week-old *Lmna^flox/flox^* and *Lmna^flox/flox^SM22αCre* mice. Lamin A/C is visualized in red, cell membranes in green (wheat germ agglutinin; WGA), cardiac fibroblasts in white (anti-FSP-1 antibody), and nuclei in blue (Hoechst 33342 staining). Graphs show the percentages of lamin A/C-positive nuclei in cardiomyocytes and cardiac fibroblasts. Statistical analysis was conducted using an unpaired two-tailed Student’s *t*-test. Data are mean ± SEM. Each symbol represents one animal.

We crossed *Lmna^flox/flox^* mice [25] with *SM22αCre* transgenic mice [26] to generate *Lmna^flox/flox^SM22αCre* mice with *Lmna* deletion restricted to VSMCs, cardiac fibroblasts, and cardiomyocytes. To examine the efficiency and specificity of lamin A/C deletion in *Lmna^flox/flox^SM22αCre* mice, we performed immunofluorescence experiments with antibodies against lamin A/C, CD31 (to detect endothelial cells), and SMA (to detect VSMCs). As expected, lamin A/C expression was robust in all cell types in the aorta, liver, kidney, and lung in control *Lmna^flox/flox^* mice (Figure 1A) but was undetectable in medial VSMCs in the vessels of *Lmna^flox/flox^SM22αCre* mice, without apparent differences in other cell types (Figure 1B). While gross examination revealed widespread and robust lamin A/C expression in heart cross-sections in *Lmna^flox/flox^* mice (Figure 2A), lamin A/C was undetectable in the coronary artery VSMCs of *Lmna^flox/flox^SM22αCre* mice and was below normal levels in non-vascular myocardial tissue (Figure 2B). To identify which cell types in the *Lmna^flox/flox^SM22αCre* myocardium had lamin A/C deficiency, heart cross-sections were co-stained with anti-lamin A/C antibodies, wheat germ agglutinin (WGA) to visualize cardiomyocyte cell membranes, and anti-fibroblast specific protein 1 (FSP-1) antibodies to detect cardiac fibroblasts. These studies revealed that lamin A/C expression was undetectable in ~87% of cardiomyocytes and ~72% of cardiac fibroblasts in *Lmna^flox/flox^SM22αCre* mice (Figure 2C).

**Figure 3:**
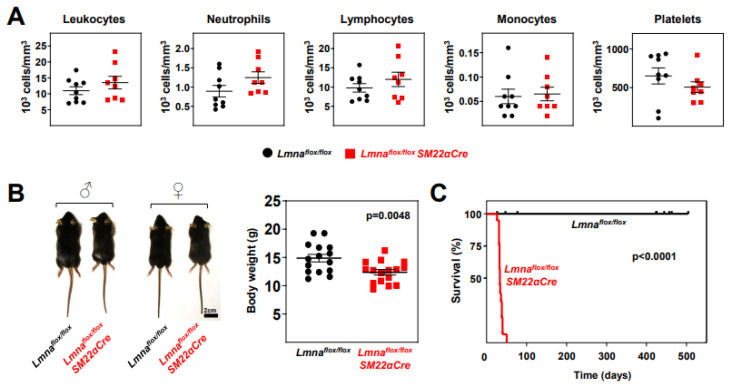
*Lmna^flox/flox^SM22αCre* mice exhibit reduced body weight and lifespan. (**A**) Circulating blood cell counts in 4-week-old *Lmna^flox/flox^* and *Lmna^flox/flox^SM22αCre* mice. (**B**) Representative photograph of male and female mice of both genotypes. The graph shows body weight at 4 weeks of age. (**C**) Kaplan–Meier survival curves of *Lmna^flox/flox^SM22αCre* (median survival 33 days) and control *Lmna^flox/flox^* mice (n = 19 mice per genotype). Statistical analysis was conducted using an unpaired two-tailed Student’s *t*-test (**A**,**B**) and the log-rank (Mantel–Cox) test (**C**). Data are mean ± SEM. Each symbol represents one animal.

Phenotypic characterization of *Lmna^flox/flox^* and *Lmna^flox/flox^SM22αCre* mice revealed no between-genotype differences in circulating blood cell populations (Figure 3A); however, *Lmna^flox/flox^SM22αCre* mice had slightly lower body weight (16.8% lower) (Figure 3B) and dramatically reduced survival, with a median lifespan of 33 days and maximum survival of 50 days (Figure 3C).

### 2.2. Lmna^flox/flox^SM22α-Cre Mice Develop Cardiac Fibrosis and Severe Systolic Dysfunction and Electrocardiographic Alterations

**Figure 4:**
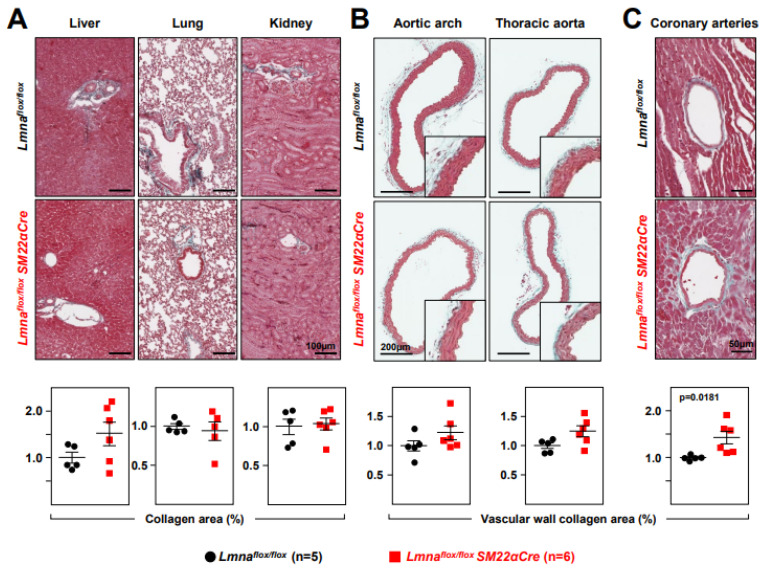
*Lmna^flox/flox^SM22αCre* mice show increased vascular collagen content. Representative images of Masson’s trichrome staining in the (**A**) liver, lung, kidney, (**B**) aortic arch, thoracic aorta, and (**C**) coronary arteries of 4-week-old *Lmna^flox/flox^* and *Lmna^flox/flox^SM22αCre* mice. Graphs show collagen content calculated relative to the content in *Lmna^flox/flox^* mice (=1). Statistical analysis was conducted using an unpaired two-tailed Student’s *t*-test. Data are mean ± SEM. Each symbol represents one animal.

**Figure 5:**
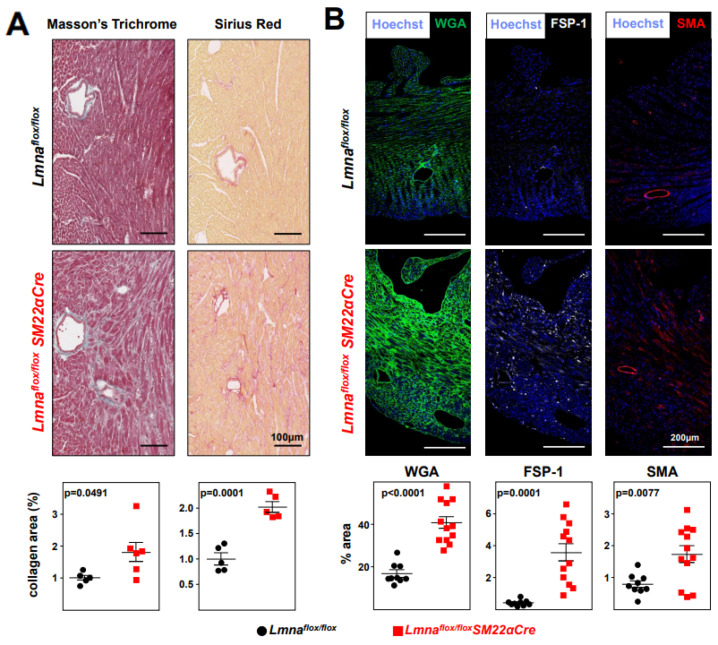
*Lmna^flox/flox^SM22αCre* mice develop cardiac fibrosis. *Lmna^flox/flox^* and *Lmna^flox/flox^SM22αCre* mice were examined at 4 weeks of age. (**A**) Representative images of Masson’s trichrome and Sirius red staining; graphs show collagen content in heart vessel-free regions calculated relative to the content in *Lmna^flox/flox^* mice (=1). One outlier identified with the GraphPad outlier calculator in the *Lmna^flox/flox^SM22αCre* group was eliminated. (**B**) Representative immunofluorescence images of heart tissue stained with wheat germ agglutinin to visualize cell membranes (WGA, green), anti-FSP-1 antibody to identify cardiac fibroblasts (white), and anti-smooth muscle actin antibody to identify fibrogenic activated fibroblasts (SMA, red). Graphs show the positive area relative to the total area of tissue. Statistical analysis was conducted using an unpaired two-tailed Student’s *t*-test. Data are mean ± SEM. Each symbol represents one animal.

**Figure 6:**
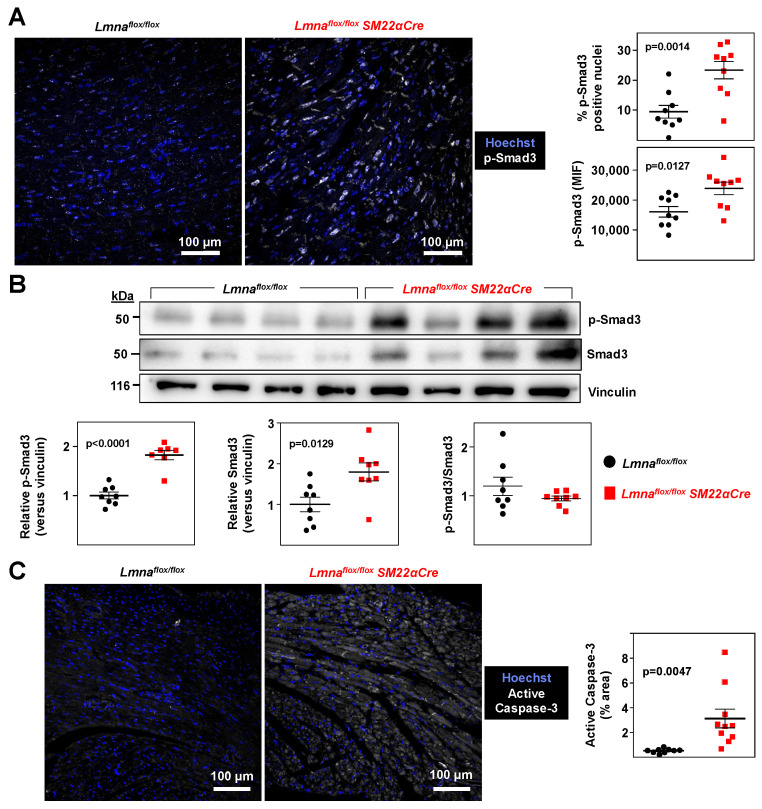
Increased total Smad3, phosphorylated Smad3, and active caspase 3 protein levels in *Lmna^flox/flox^SM22αCre* mouse hearts. *Lmna^flox/flox^* and *Lmna^flox/flox^SM22αCre* mice were examined at 4 weeks of age. (**A**) Representative immunofluorescence images of heart tissue showing phosphorylated Smad3 (S423 + S425; p-Smad3; white) and nuclei (stained with Hoechst 33342; blue). Graphs show p-Smad3-positive nuclei (top) and median intensity fluorescence (MIF) of p-Smad3-positive nuclei (bottom). (**B**) Representative Western blots of heart protein lysates probed with antibodies against p-Smad3, Smad3, and vinculin (the latter used as a housekeeping loading control). Each lane corresponds to the heart tissue from one mouse. Membranes incubated with anti-p-Smad3 antibody were stripped off and used for incubation with anti-Smad3 antibody. Graphs show relative p-Smad3 and total Smad3 expression normalized to vinculin expression (left and middle graphs, respectively) and the p-Smad3/Smad3 ratio after normalization to vinculin (right graph). One outlier identified in the *Lmna^flox/flox^SM22αCre* group using the Grubbs’ test was not included in the left graph. (**C**) Representative immunofluorescence images of the active (cleaved) form of caspase-3 (white) and nuclei (Hoechst 33342; blue). The graph shows the active caspase-3 positive area as a percentage of the total area of tissue. Statistical analysis was conducted using an unpaired two-tailed Student’s *t*-test. Data are mean ± SEM. Each symbol represents one animal.

Immunohistopathological studies in 4-week-old *Lmna^flox/flox^SM22αCre* mice and *Lmna^flox/flox^* controls showed no between-genotype differences in collagen content in tissue sections from the liver, lung, and kidney (Figure 4A). However, *Lmna^flox/flox^SM22αCre* mice showed a trend towards increased collagen content in the aortic arch and thoracic aorta (Figure 4B), which reached statistical significance in coronary arteries (Figure 4C). Moreover, *Lmna^flox/flox^SM22αCre* hearts had significantly higher interstitial fibrosis (Figure 5A), elevated WGA staining, and above-normal expression of the profibrotic markers FSP-1 and SMA (Figure 5B). These alterations in mutant hearts were associated with a higher expression of p-Smad3, the active form of the pro-fibrotic transcription factor Smad3, as revealed by immunofluorescence experiments (Figure 6A). Western blot analysis confirmed an elevated expression of p-Smad3 in *Lmna^flox/flox^SM22αCre* hearts, which was accompanied by a higher expression of total Smad3 without changes in the p-Smad3/Smad3 ratio (Figure 6B, left, middle, and right graphs, respectively). *Lmna^flox/flox^SM22αCre* hearts also had a higher expression of the active (cleaved) form of the pro-apoptotic protein caspase-3 (Figure 6C).

**Figure 7:**
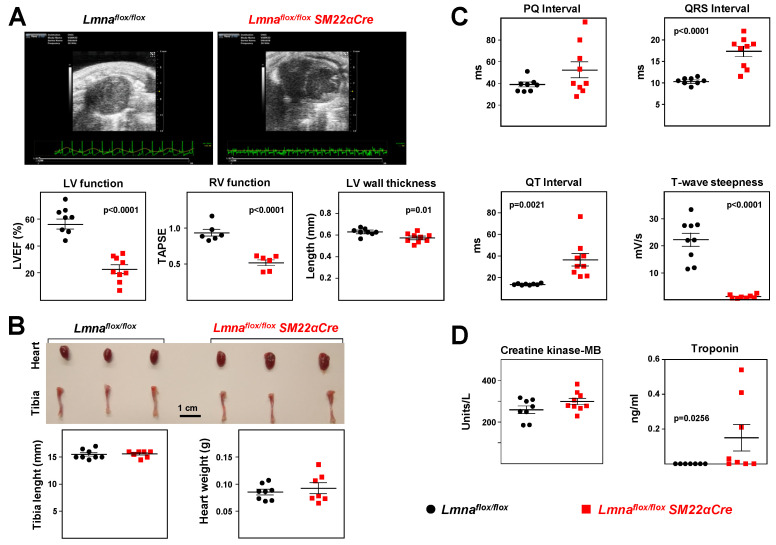
*Lmna^flox/flox^SM22αCre* mice show a severe loss of cardiac function and electrocardiographic defects. *Lmna^flox/flox^* and *Lmna^flox/flox^SM22αCre* mice were examined at 4 weeks of age. (**A**) Representative echocardiography images (sagittal plane) and quantification of left ventricle (LV) function (EF, ejection fraction), right ventricle (RV) function (TAPSE, tricuspid annular plane systolic excursion), and LV wall thickness. These results are also shown in table format in Supplementary Table S1. Sagittal and longitudinal planes are shown in Supplementary Videos S1–S4. (**B**) Representative images of hearts and tibia bones and quantification of tibia length and heart weight. (**C**) Quantification of PQ, QRS, and QT intervals and T-wave steepness obtained by electrocardiography. (**D**) Plasma levels of creatine kinase MB isoform (CK-MB) and troponin. One outlier identified in the *Lmna^flox/flox^SM22αCre* group using the Grubbs’ test was not included in the analysis of plasma troponin. Statistical analysis was conducted using an unpaired two-tailed Student’s *t*-test for CK-MB and by the non-parametric Mann–Whitney test for troponin (troponin data did not follow a normal distribution). Data are mean ± SEM. Each symbol represents one animal.

Echocardiography analysis detected significant systolic dysfunction in both the left and right ventricles of *Lmna^flox/flox^SM22αCre* mice, revealed by lower EF and TAPSE, respectively (Figure 7A, Supplementary Table S1, and Supplementary Videos S1–S4). *Lmna^flox/flox^SM22αCre* mice also showed a modest but statistically significant decrease in left ventricle mass thickness (Figure 7A), whereas heart weight and tibia length were similar in both genotypes (Figure 7B). ECG analysis revealed statistically significant between-genotype differences in parameters indicative of a lower repolarization rate in *Lmna^flox/flox^SM22αCre* mice, including prolongation of the QRS and QT intervals and reduced T-wave steepness (Figure 7C). We found no between-genotype differences in plasma levels of creatine kinase-MB and significantly elevated plasma troponin in *Lmna^flox/flox^SM22α-Cre* mice, despite high interindividual variability in mutant mice (Figure 7D).

### 2.3. Lmna^flox/flox^SM22αCre Mice Exhibit Contractile-to-Synthetic Phenotypic Switching in VSMCs and Vascular Dysfunction in the Aorta

**Figure 8:**
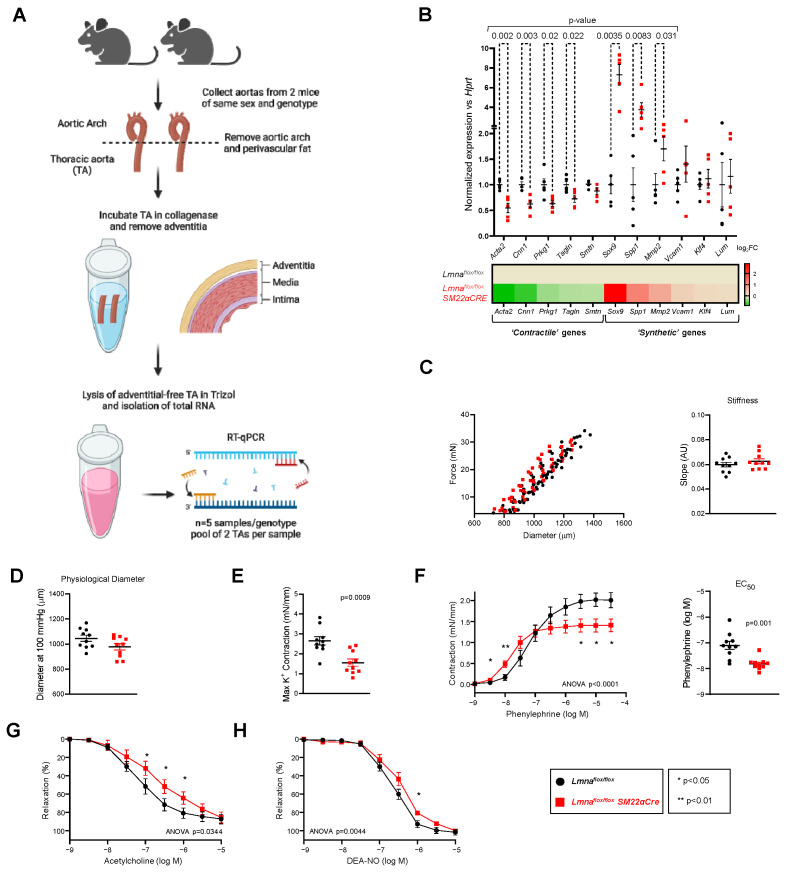
*Lmna^flox/flox^SM22αCre* mice exhibit contractile-to-synthetic phenotypic switching in vascular smooth muscle cells and vascular dysfunction in the aorta. *Lmna^flox/flox^* and *Lmna^flox/flox^SM22αCre* mice were examined at 4 weeks of age. (**A**) Protocol for processing mouse thoracic aorta (TA) samples for real-time quantitative PCR (RT-qPCR). Aortas were isolated from *Lmna^flox/flox^* and *Lmna^flox/flox^SM22αCre* mice. After removing the aortic arch and perivascular tissue, TA samples were incubated with type I collagenase. Adventitia was then removed manually, and samples from mice of the same genotype and sex were paired for isolation of total RNA. Created with BioRender.com. (**B**) RT-qPCR analysis of adventitia-free thoracic aorta, examining the expression of vascular smooth muscle cell ‘contractile’ and ‘synthetic’ genes (n = 5). Each replicate contained the thoracic aortas from two mice of the same genotype and sex. Data are presented as the ΔCt fold change relative to control samples. *Hprt* was used as the housekeeping gene. The heatmap shows the log_2_ of the fold change relative to control *Lmna^flox/flox^* mice. (**C**–**H**) Thoracic aorta rings were mounted in a wire myograph system to examine the following parameters (n = 10 each genotype): diameter–force relationship and its linear regression slope (an estimation of vessel stiffness) (**C**); estimated aortic ring diameter at 100 mmHg (**D**); maximum response induced by 120 mmol/L KCl (**E**); concentration–response curves to phenylephrine, and the concentration of phenylephrine giving the half-maximal response (EC_50_) (**F**); endothelium-dependent vasodilation induced by increasing concentrations of acetylcholine; (**G**); and endothelium-independent vasodilation induced by increasing concentrations of diethylamine NONOate (DEA-NO) (**H**). Statistical differences were analyzed using an unpaired two-tailed Student’s *t*-test in (**B**,**C**) (right graph) and (**D**–**F**) (right graph) or by two-way ANOVA and Fisher’s LSD multiple comparisons test in the dose–response curves in (**F**–**H**). Data are mean ± SEM.

To investigate possible alterations in the vasculature of *Lmna^flox/flox^SM22αCre* mice, we performed RT-qPCR on total RNA isolated from adventitia-free aortic tissue (Figure 8A). These studies revealed significant downregulation in the expression of genes characteristic of ‘contractile’ VSMCs and upregulation of markers of ‘synthetic’ VSMCs in *Lmna^flox/flox^SM22αCre* mice (Figure 8B), with no between-genotype differences in other genes relevant to VSMC function, including genes related to calcium homeostasis (*Cam2*), oxidative stress (*Nox1, Sod1*), and mitochondrial and sarcoplasmic reticulum function (*Tfam, Calr*) (Supplementary Figure S1).

Ex vivo wire myography experiments with thoracic aorta rings revealed no between-genotype differences in vessel stiffness (Figure 8C) or physiological diameter (Figure 8D). However, the potassium-stimulated maximum contraction was significantly lower in *Lmna^flox/flox^SM22αCre* mice (Figure 8E). Likewise, although aortic rings with VSMC-specific *Lmna* ablation contracted more than controls at phenylephrine doses below 10^−7^ M, their maximum contraction was lower at higher phenylephrine concentrations, with a significantly lower EC_50_ (phenylephrine dose giving a half-maximal response) (Figure 8F). Interestingly, a lack of lamin A/C in aortic VSMCs was also associated with significantly lower endothelium-dependent vasorelaxation induced by acetylcholine (Figure 8G) and endothelium-independent vasorelaxation induced by the NO donor DEA-NO (Figure 8H).

## 3. Discussion

The major severe clinical manifestations of *LMNA*-DCM are sudden cardiac death and progressive LVEF deterioration [10,11,12,13,14,15,16]. Therefore, most studies have sought to define the mechanisms through which *LMNA* mutations provoke cardiac alterations, with a particular focus on cardiomyocytes. However, A-type lamins are expressed in most differentiated cells, and it is therefore of the utmost interest to investigate the potential pathological effects of *LMNA* mutations on other cell types, which may cause alterations in cardiac muscle through paracrine mechanisms. In the present study, we generated and characterized *Lmna^flox/flox^SM22αCre* mice with constitutive lamin A/C deficiency in VSMCs, cardiac fibroblasts, and cardiomyocytes, abundant cell types in the heart. Consistent with previous studies in *Myh6-Cre:Lmna^f/f^* mice with lamin A/C deficiency exclusively in cardiomyocytes [22,23], *Lmna^flox/flox^SM22αCre* mice recapitulated the main hallmarks of human *LMNA*-DCM, including cardiac fibrosis, ventricular systolic dysfunction, ECG alterations, and premature death. Importantly, the time course and severity of disease in *Lmna^flox/flox^SM22αCre* mice were strikingly similar to observations in whole-body *Lmna-null* mice and the cardiomyocyte-specific *Myh6-Cre:Lmna^f/f^* mice [19,22,23]. Indeed, our new model demonstrates that extending lamin A/C disruption to VSMCs and cardiac fibroblasts does not aggravate the lifespan reduction caused by *Lmna* deficiency restricted to cardiomyocytes (median lifespan ~1 month in both models). Recent studies in *Pdfra-Cre:Lmna^f/f^* mice with lamin A/C absence in ~80% of cardiac fibroblasts and ~25% of cardiomyocytes partially recapitulated the *LMNA*-DCM phenotype, with a median lifespan of ~43 days [24]. Moreover, restoration of lamin A expression in ~40% of cardiomyocytes in *Lmna^−/−^; Tg* mice partially rescued ECG alterations and extended lifespan by 12% compared with controls with whole body *Lmna* ablation [21]. Collectively, the results in these mouse models suggest that cardiomyocyte-autonomous and non-cardiomyocyte-autonomous factors play an important role in the etiopathogenesis of *LMNA*-DCM. Further discrimination of the individual role of VSMCs and cardiac fibroblasts in *LMNA*-DCM would require the generation of new mouse models with *Lmna* deficiency restricted to these cell types.

Consistent with previous studies in *Lmna^−/−^* and *Myh6-Cre:Lmna^f/f^* mice [22,23], *Lmna^flox/flox^SM22αCre* mice showed evidence of cardiac fibrosis and apoptosis, which was accompanied by elevated fibroblast and myofibroblast markers, including WGA staining and immunostaining of FSP-1 and SMA. Previous studies have suggested that cardiomyocyte apoptosis in *Lmna*-null mice may result from altered gene expression, disruption of cytoskeleton tension, and defective force transmission [20]. Regarding fibrosis, it is well-known that members of the transforming growth factor β (TGFβ) superfamily trigger pro-fibrotic transcriptional programs through the activation of SMAD-dependent signaling in cardiomyocytes, fibroblasts, immune cells, and vascular cells [27]. For example, phosphorylation of SMAD3 triggers the conversion of cardiac fibroblasts into secretory pro-fibrotic myofibroblasts expressing extracellular matrix and contractile proteins (such as SMA) and integrins, thus promoting myofibroblast migration, survival, and growth arrest and scar formation [27]. We found elevated Smad3 and p-Smad3 expression in *Lmna^flox/flox^SM22αCre* hearts without changes in the p-Smad3/Smad3 ratio. Phosphorylation (activation) of Smad proteins was also observed in the hearts of *Lmna^H222P/H222P^* mice [28], and *Lmna*-null cardiomyocytes isolated from *Lmna^flox/flox^Myh6-Cre* mice exhibited high TGFβ1 mRNA and protein expression [22]. Collectively, these results suggest that lamin A/C deficiency and expression of the DCM-causing *Lmna^H222P^* protein provoke cardiac fibrosis at least in part through activation of TGFβ/SMAD signaling. Future studies are warranted to assess if TGFβ/SMAD signaling is abnormally activated in the heart in other mouse models of *LMNA*-DCM and in patients.

Uncertainty has surrounded the question of whether vascular abnormalities exist and play a role in the pathogenesis and progression of non-ischemic DCM [29]. Mathier et al. reported the presence of abnormal coronary endothelium-dependent vasodilation in the epicardium and the microcirculation at early disease stages in patients with acute-onset DCM [30]. These authors also found an association between the preservation of endothelial function and improved LVEF in this population [30]. Furthermore, non-ischemic DCM has been linked to vascular derangements and to defective vasculogenesis and angiogenesis in patients [29]. Recently, Sayed et al. reported clinical endothelial dysfunction in patients with *LMNA*-DCM, and human induced pluripotent stem cell-derived endothelial cells (ECs) carrying DCM-causing *LMNA* mutations presented hallmarks of endothelial dysfunction, including a decreased capacity to produce nitric oxide and impaired angiogenic potential in vitro [31]. To our knowledge, our current results provide the first evidence that *Lmna* deficiency also provokes VSMC alterations in vivo. Four-week-old *Lmna^flox/flox^SM22αCre* mice displayed a phenotype switch in the aorta from the ‘contractile’ phenotype characteristic of ‘healthy’ VSMCs to the ‘synthetic’ VSMC phenotype that characterizes the inflamed vessel wall in various forms of vascular disease [32]. The mice also showed incipient fibrosis in the aorta, which reached statistical significance in coronary arteries. Moreover, our myograph studies show that aortic rings with VSMC-specific *Lmna* deficiency have impaired maximum contraction and a defective response to vasodilators and vasoconstrictors with accompanying alterations in relaxation induced by the nitric oxide donor DEA-NO, evidencing VSMC dysfunction. VSMC injury might also contribute to the endothelial dysfunction reported in *LMNA*-DCM patients [31] since aortic rings lacking lamin A/C specifically in VSMCs had depressed endothelium-dependent acetylcholine-induced relaxation despite normal lamin A/C expression in ECs. Alterations in gene expression and function in lamin A/C-deficient VSMCs and ECs are likely caused by defective mechanotransduction and altered signaling, transcription, and chromatin organization, which are key processes that are regulated by A-type lamins [4,6]. Collectively, these studies suggest that dysfunctional ECs and VSMCs contribute to the etiopathogenesis of *LMNA*-DCM and that therapies to ameliorate vascular cell function may have a beneficial effect on the heart. Consistent with this, treatment with lovastatin ameliorated endothelial function in cultured *LMNA* iPSC-ECs and in *LMNA*-DCM patients and also improved the functional phenotype of *LMNA* iPSC-derived cardiomyocytes when co-cultured with *LMNA* iPSC-ECs [31]. Although mouse models harboring cell-type-specific *Lmna* alterations have provided important insights into the mechanisms underlying *LMNA*-DCM, future mechanistic and therapeutic studies should use more translational ubiquitous models that consider the crosstalk among cardiac and non-cardiac cells. More research is also warranted to identify the mechanisms that cause vascular dysfunction in *LMNA*-DCM and to investigate vascular pathology in *LMNA*-DCM patients, as these approaches may open new therapeutic avenues for the treatment of these diseases.

## 4. Materials and Methods

### 4.1. Mice

*Lmna^flox/flox^* mice [25] and *SM22αCre* mice (*TaglnCre*, The Jackson Laboratory, stock no: 017491) [26], both on the C57BL/6J genetic background, were crossed to generate *Lmna^flox/flox^SM22Cre* mice. Genotyping was performed by PCR analysis of genomic DNA extracted from mouse tails using the primers shown in Table 1. All experiments were performed with 4-week-old mice and balanced numbers of males and females.

### 4.2. Longevity Studies

Animals were weighed periodically and inspected daily for health status and survival by a veterinarian blinded to genotype. Animals that met humane end-point criteria were euthanized and censored in the Kaplan–Meier survival analysis. Animals sacrificed due to hydrocephalus, malocclusion, inter-male aggression, or other reasons unconnected to phenotype were excluded from the analysis.

### 4.3. Hematology and Cardiac Biochemical Parameters

Blood was extracted from the submaxillary vein and collected in microvette 100 K3 EDTA tubes (Sarstedt, Nümbrecht, Germany), and circulating blood cell populations were quantified using the PENTRA 80 hematology platform (HORIBA Medical, Madrid, Spain). Plasma was isolated by centrifugation of whole blood (2000× *g*, 15 min at room temperature). Creatine kinase-MB and troponin were measured in plasma using a DIMENSION RxL MAX chemistry analyzer (Siemens, Munich, Germany).

### 4.4. Histology and Immunofluorescence

All mouse organs were fixed in 4% paraformaldehyde for 48 h, dehydrated through 50%, 70%, 95%, and 100% alcohol, embedded in paraffin, and cut in 5 μm sections using a HM 355S microtome (Thermo Scientific, Waltham, MA, USA). For immunofluorescence analysis of active caspase-3 and phospho-Smad 3 (S423 + S425) (p-Smad 3) on heart sections, antigens were retrieved with 10 mM sodium citrate buffer (pH6) or TRIZMA base EDTA (pH9). Samples were then blocked and permeabilized for 1 h at room temperature in PBS containing 0.3% Triton X100 (9002-93-1, Sigma, Kawasaki, Japan), 5% bovine serum albumin (BSA, A7906, Sigma), and 5% normal goat serum (005-000-001, Jackson ImmunoResearch, West Grove, PA, USA). Sections were then incubated overnight at 4 °C with antibodies against CD31 (ab28364, Abcam, Cambridge, UK, 1:100), lamin A/C (sc-376248, Santacruz, Santa Cruz, CA, USA, 1:100), FSP-1 (A5114, Dako, Glostrup, Denmark, 1:200), the active (cleaved) form of caspase-3 (AF835, R&D Systems, Minneapolis, MN, USA, 1:200; distinguishes apoptotic cells from non-apoptotic cells), and p-Smad 3 (S423 + S425) [EP823Y] (ab52903, Abcam, 1:100). After washes, sections were incubated for 2 h at room temperature with an antibody to smooth muscle α-actin (SMA) conjugated to Cy3 (C6198, Sigma, 1:20), anti-rabbit-Alexa Fluor 647 secondary antibody (111-607-008, Jackson ImmunoResearch, 1:400), wheat germ agglutinin-Alexa Fluor 488 (W11261, ThermoFisher, Waltham, MA, USA, 1:300), and Hoechst 33342 or DAPI (1:1000). Sections were mounted in Fluoromount-G imaging medium (00-4958-02, ThermoFisher).

For histological studies, tissue sections were stained with hematoxylin/eosin, picrosirius red, or Masson’s trichrome using standard protocols. Images were scanned with a NanoZoomer-RS scanner (Hamamatsu, Shizuoka, Japan) and were exported using NDP.view2. Immunofluorescence images were acquired with a Zeiss LSM700 confocal microscope. Images were analyzed with ImageJ software version 1.53c by an operator blinded to genotype. At least 3 tissue sections per animal were analyzed, with the removal of the immunofluorescence signals from arterioles. Mean values were used for statistical analysis. 

### 4.5. Aortic RNA Extraction and Real-Time Quantitative PCR (RT-qPCR)

Mouse thoracic aortas were incubated for 10 min at 37 °C in DMEM (Gibco, Billings, MT, USA) containing 2 mg/mL type I collagenase (Worthington, Columbus, OH, USA, LS004194). Adventitia was then removed manually, and the remaining tissue was snap-frozen and stored at −80 °C until further use. Each biological sample was a pool of two aortas from mice of the same genotype and sex. RNA was isolated using the RNeasy Mini Kit (Qiagen, Hilden, Germany). RNA (1 μg) was reverse-transcribed to cDNA using the High Capacity cDNA Reverse Transcription kit (Applied Biosystems, Waltham, MA, USA). Real-time quantitative PCR (RT-qPCR) was performed using the primers shown in Table 2 and Power SYBR Green PCR Master Mix (Applied Biosystems) in a C1000 Touch Thermal Cycler (Bio-Rad, Hercules, CA, USA). All the values were normalized to the housekeeping hypoxanthine-guanine phosphoribosyl transferase (HPRT) gene. All reactions were performed in triplicate.

### 4.6. Western Blot

Snap-frozen hearts were lysed in ice-cold 50 mM Tris-HCl buffer (pH 8.8) containing 2% SDS, 8 M Urea, and 2 M thiourea using a TissueLyser (Qiagen). Lysates (25 μg protein) were mixed with loading buffer including 11 mg/mL 2-mercaptoethanol, incubated at 95 °C for 5 min, and resolved on SDS-10% polyacrylamide gels. Proteins were transferred to a PVDF membrane (Immobilon-P pore 0.45 μm, Sigma Aldrich, St. Louis, MO, USA) using standard methods for wet transfer. Membranes were blocked for 1 h in 5% BSA-TBS-T for anti-p-Smad3 and 5% milk-TBS-T for the rest of the antibodies (TBS-T: Tris-buffered saline supplemented with 0.2% Tween-20). Membranes were incubated overnight at 4 °C with the following primary antibodies: anti-Smad3 (EP568Y) (ab40854, Abcam, 1:1000), anti-phospho-Smad3 (S423 + S425) (EP823Y) (ab52903, Abcam, 1:1000), and anti-Vinculin Clone hVIN-1 (V9131, Sigma, 1:1000). After extensive washes with TBST-T, immunocomplexes in the membranes were detected with species-appropriate HRP-conjugated secondary antibodies and were visualized using HRP Western Luminata Forte (WBLUF0100, Millipore, Burlington, MA, USA). The relative intensity of protein bands was determined by densitometry with ImageQuant software version 1.2.

### 4.7. Echocardiography

Transthoracic echocardiography was performed by expert operators using a high-frequency ultrasound system (Vevo 2100, Visualsonics Inc., Toronto, ON, Canada) with a 40 MHz linear probe. Operators were blinded to genotype. Two-dimensional (2D) and M-mode (MM) echography scans were performed at a frame rate > 230 frames/sec, and pulse wave Doppler (PW) was acquired with a pulse repetition frequency of 40 kHz. Mice were anaesthetized with 0.5–2% isoflurane in oxygen, with isoflurane delivery adjusted to maintain the heart rate at 450 ± 50 beats per minute (bpm). Mice were placed in a supine position on a heating platform, warmed ultrasound gel was used to maintain normothermia, and eye-drop gel was used to prevent dryness. A base apex electrocardiogram (ECG) was continuously monitored. Images were analyzed off-line using the Vevo 2100 Workstation software v5.6.1. For left ventricular systolic function assessment, parasternal standard 2D and MM long and short axis views were acquired. Left ventricular ejection fraction (LVEF), fractional shortening, stroke volume, cardiac output, and thickness were calculated from these views. Right ventricular systolic function was indirectly estimated using tricuspid annular plane systolic excursion (TAPSE), obtained from a MM 4-chamber apical view, to measure maximum lateral tricuspid annulus movement. 

### 4.8. Electrocardiography

Mice were anaesthetized with 0.5–1.5% isoflurane in oxygen. To avoid night–day circadian variations, ECGs were always recorded in the morning. ECG electrodes were inserted subcutaneously in the four limbs. Sequential ECG recordings were acquired at 2 kHz using an MP36R data acquisition workstation (Biopac Systems, Goleta, CA, USA) and exported with AcqKnowledge software v4.1 (Biopac Systems). Automatic analysis with custom R scripts was used to remove noise and baseline fluctuations; detect heartbeat, peaks, and waves; exclude artifacts; and calculate heart rate, QRS and QT intervals, and T-wave steepness. Lead II was selected for the study, since the signal was more stable in most experiments, allowing more robust wave identification. Heart rate was calculated as the inverse of the time difference between two consecutive R-wave peaks (RR). Due to potential noise interference with the detection of the beginning of P-waves, the PQ interval was measured from the P-wave peak to the beginning of the Q-wave. The end of the S-wave (J) is not evident in mice because the ST segment is absent in this species and is replaced by a J-wave corresponding to a positive segment of the T-wave. Therefore, the QRS complex was calculated from the Qs to the S-wave minimum and the QT interval from the Qs to the T-wave peak (also termed J-wave peak). T-wave morphological alterations were quantified by defining T-wave steepness as an indicator of T-wave flattening that represents the absolute value of the slope (voltage variation over time) between the T-wave peak and T_90_.

### 4.9. Wire Myography

Aortas were dissected free of fat and connective tissue and placed in cold Krebs Henseleit Solution (KHS: 115 mmol/L NaCl, 25 mmol/L NaHCO_3_, 4.7 mmol/L KCl, 1.2 mmol/L MgSO_4_·7H_2_O, 2.5 mmol/L CaCl_2_, 1.2 mmol/L KH_2_PO_4_, 11.1 mmol/L glucose, and 0.027 mmol/L EDTA). Segments of thoracic aortas, 2 mm in length, were mounted on a wire myograph system (620 M, DMT) for isometric tension recording. After a 30 min equilibration period in KHS oxygenated with a mixture of 95% O_2_ and 5% CO_2_ at 37 °C and pH 7.4, diameter–tension relationships were determined by increasing the distance between the wires passing through the lumen, thus increasing its passive diameter. At each step, the force and the internal circumference of the vessel were recorded [33]. Then, segments were stretched to their optimal lumen diameter for active tension development (LabChart software, ADInstruments, Sydney, Australia, [33]). This was determined based on the internal circumference/wall tension ratio of the segments by setting their internal circumference, Lo, to 90% of what the vessels would have if they were exposed to a passive tension, which is equivalent to that produced by a transmural pressure of 100 mm Hg. 

The contractility of the segments was tested by an initial exposure to a high K^+^ solution (K^+^-KHS, 120 mmol/L). After an equilibration period, aortic segments were precontracted with phenylephrine at ∼50% K^+^-KHS contraction in order to perform a concentration–response curve to acetylcholine (1 nmol/L–10 µmol/L). After washing, a concentration–response curve to phenylephrine (1 nmol/L–30 µmol/L) was performed. Finally, a concentration–response curve to diethylamine NONOate (DEA-NO, 1 nmol/L–10 µmol/L) was performed in phenylephrine pre-contracted arteries.

### 4.10. Statistical Analysis

Quantitative data are presented as the mean ± the standard error of the mean (SEM). Statistical analysis was performed with GraphPad Prism. The normal distribution of the data was analyzed using the Kolmogorov–Smirnov test. The statistical significance of differences was assessed as indicated in the figure legends. Differences were considered significant at *p*-values < 0.05. Outliers identified using the Grubbs’ test in the GraphPad outlier calculator were eliminated as indicated in the legends of figures.

## Figures and Tables

**Table 1:** Primer sequences used for mouse genotyping.

Genotyping	Forward (5′ → 3′)	Reverse (5′ → 3′)
*SM22αCre* Transgene	GCGGTCTGGCAGTAAAAACTATC	GTGAAACAGCATTGCTGTCACTT
*SM22αCre* Internal positive control	CTAGGCCACAGAATTGAAAGATCT	GTAGGTGGAAATTCTAGCATCATCC
*Lmna^flox/flox^*	AACCCAGCCTCAGAAACTGGTGGATG	GACAGCTCTCCTCTGAAGTGCTTGGA

**Table 2:** Primer sequences used for real-time quantitative PCR.

Gene	Forward (5′ → 3′)	Reverse (5′ → 3′)
*Acta2*	AAGAGGAAGACAGCACAGCC	AGCGTCAGGATCCCTCTCTT
*Calr*	CCAGAAATTGACAACCCTGAA	CCTTAAGCCTCTGCTCCTCAT
*Cam2*	AAGTTGATGAAATGATCAGGGAAG	TGAAGTCCTAATTACTATACATGCATA
*Cnn1*	TGGGAGTCAAGTATGCAGAG	CTGACTGGCAAACTTGTTGG
*Hprt*	CCTAAGATGAGCGCAAGTTGAA	CCACAGGACTAGAACACCTGCTAA
*Klf4*	TTGTGACTATGCAGGCTGTG	TAGTGCCTGGTCAGTTCATC
*Lum*	TTCACTGGGCTGCAATACC	TCCCAGGATCTTACAGAAGC
*Mmp2*	ACCTTGACCAGAACACCATC	AGCATCATCCACGGTTTCAG
*Nox1*	CAACAGCACTCACCAATGCC	ACATCCTCACTGACTGTGCC
*Prkg1*	ACTGCATGTGTGGTAGAAGC	GCCAGTCAGAAGCTCATACATC
*Smtn*	AGAACACCATCACCCACATC	TCTTGTCCAGGACTCCTTCG
*Sod1*	TGGGTTCCACGTCCATCAGTA	ACCGTCCTTTCCAGCAGTCA
*Sox9*	AGAACAAGCCACACGTCAAG	GTCTCTTCTCGCTCTCGTTC
*Spp1*	GGTGATAGCTTGGCTTATGG	TGGGCAACAGGGATGACATC
*Tagln*	CCCAGACACCGAAGCTACTC	GACTGCACTTCTCGGCTCAT
*Tfam*	CAGGAGGCAAAGGATGATTC	CCAAGACTTCATTTCATTGTCG
*Vcam1*	TCAAGGGTGACCAGCTCATG	TCGTTGTATTCCTGGGAGAG

## Data Availability

Data supporting the reported results are contained within the article.

## Supplementary Materials

The supporting information can be downloaded at: https://www.mdpi.com/article/10.3390/ijms241311172/s1.
