# Supplementary material for: Lamin A/C Ablation Restricted to Vascular Smooth Muscle Cells, Cardiomyocytes, and Cardiac Fibroblasts Causes Cardiac and Vascular Dysfunction
Source: Int J Mol Sci. 2023 Jul 6;24(13):11172. doi: 10.3390/ijms241311172 (PMC10342548; doi:10.3390/ijms241311172)
Supplement: Supplementary file 1 [file ijms-24-11172-s001.zip › del Monte-Monge_supplementary material.pdf]

SUPPLEMENTARY MATERIAL

Lamin A/C ablation restricted to vascular smooth muscle cells, cardiomyocytes, and cardiac fibroblasts causes cardiac and vascular dysfunction

Alberto Del Monte-Monge[1,2], Íñigo Ruiz-Polo de Lara[1], Pilar Gonzalo[1,2], Carla Espinós-Estévez[1,2], María González-Amor[1,2], Miguel de la Fuente-Pérez[1], María J. Andrés-Manzano[1,2], Víctor Fanjul[1,2,†], Juan R. Gimeno[2,3], Roberto Barriales-Villa[2,4], Beatriz Dorado[1,2], Vicente Andrés[1,2,*]

[1] Centro Nacional de Investigaciones Cardiovasculares (CNIC), 28029 Madrid, Spain

[2] Centro de Investigación Biomédica en Red de Enfermedades Cardiovasculares (CIBERCV), 28029 Madrid, Spain

[3] Cardiac Department, Hospital Clínico Universitario Virgen Arrixaca, 30120 Murcia, Spain

[4] Unidad de Cardiopatías Familiares, Complexo Hospitalario Universitario A Coruña (INIBIC-CHUAC), 15006 A Coruña, Spain

† Present address: Savana Medical, 28013 Madrid, Spain

* Corresponding author:

Vicente Andrés

CNIC, Melchor Fernández Almagro 3, 28029 Madrid (Spain)

Phone: +34-91 453 12 00 (Ext. 1502)

E-mail: vandres@cnic.es

INDEX SUPPLEMENTARY MATERIAL

- **Supplementary figure S1. Real time quantitative PCR analysis of adventitia-free mouse thoracic aorta.**
- **Supplementary Table S1. Echocardiographic parameters in 4-week-old *Lmna$^{flox/flox}$* and *Lmna$^{flox/flox}$SM22αCre* mice.**
- **Supplementary video S1. Representative echocardiography video (sagittal plane) of a 4-week-old *Lmna$^{flox/flox}$* mouse.**
- **Supplementary video S2. Representative echocardiography video (sagittal plane) of a 4-week-old *Lmna$^{flox/flox}$SM22αCre* mouse.**
- **Supplementary video S3. Representative echocardiography video (longitudinal plane) of a 4-week-old *Lmna$^{flox/flox}$* mouse.**
- **Supplementary video S4. Representative echocardiography video (longitudinal plane) of a 4-week-old *Lmna$^{flox/flox}$SM22αCre* mouse.**

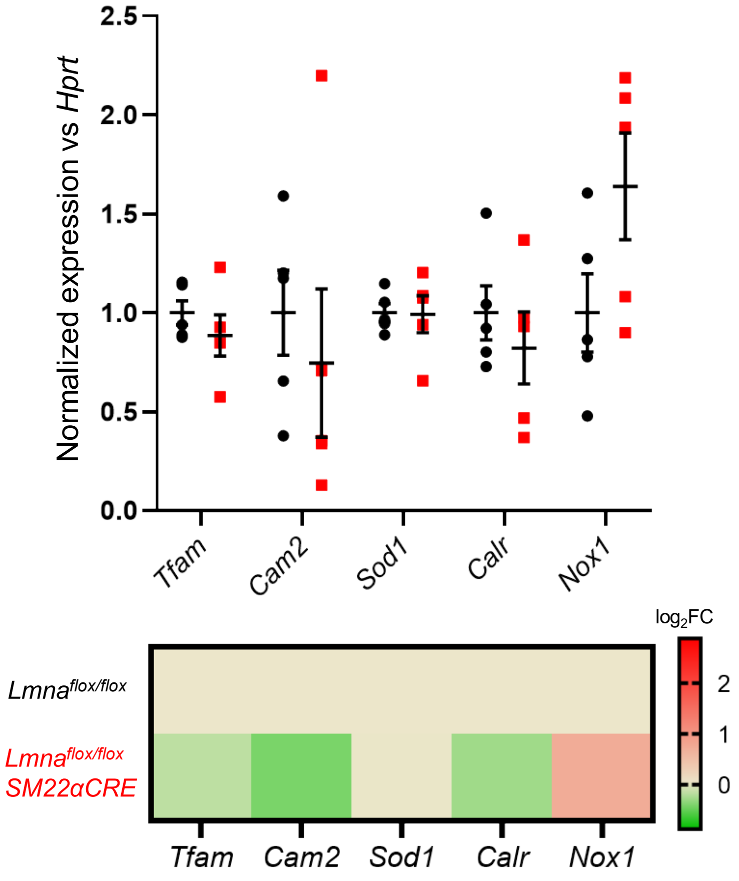

Supplementary Figure S1. Real time quantitative PCR analysis of adventitia-free mouse thoracic aorta. Aortas were isolated from *Lmna$^{flox/flox}$* and *Lmna$^{flox/flox}$SM22αCre* mice and processed as indicated in Figure 8A. The graph shows the results of real-time quantitative PCR (RT-qPCR) analysis of adventitia-free thoracic aorta, examining the expression of the indicated genes (n=5 each genotype). Data are presented as the ΔCt fold change relative to control *Lmna$^{flox/flox}$* samples. *Hprt* was used as the housekeeping gene for normalization. The heatmap shows the $\log_2$ of the fold-change relative to control *Lmna$^{flox/flox}$* mice. (=0). Statistical differences were analyzed using unpaired two-tailed Student's t-test.

Supplementary Table S1. Echocardiographic parameters in 4-week-old *Lmna*$^{flox/flox}$ and *Lmna*$^{flox/flox}$*SM22αCre* mice.

	Lmna$^{flox/flox}$	*Lmna*$^{flox/flox}$*SM22αCre*	**P value**
**Number of mice**	8	9	
**LVEF (%)**	59.43±3.519	22.76±3.103	<0.0001
**TAPSE**	0.9377±0.0509	0.05239±0.04204	<0.0001
**LVWT**	0.6297±0.01137	0.5751±0.01423	0.01

The table shows the results plotted in Figure 7A. Data are represented as the mean±SEM.

Abbreviations: LVEF, left ventricle ejection fraction; TAPSE, tricuspid annular plane systolic excursion; LVWT, left ventricle wall thickness.
