# Supplementary figures and images for: Lamin A/C Ablation Restricted to Vascular Smooth Muscle Cells, Cardiomyocytes, and Cardiac Fibroblasts Causes Cardiac and Vascular Dysfunction
Source: Int J Mol Sci. 2023 Jul 6;24(13):11172. doi: 10.3390/ijms241311172 (PMC10342548; doi:10.3390/ijms241311172)

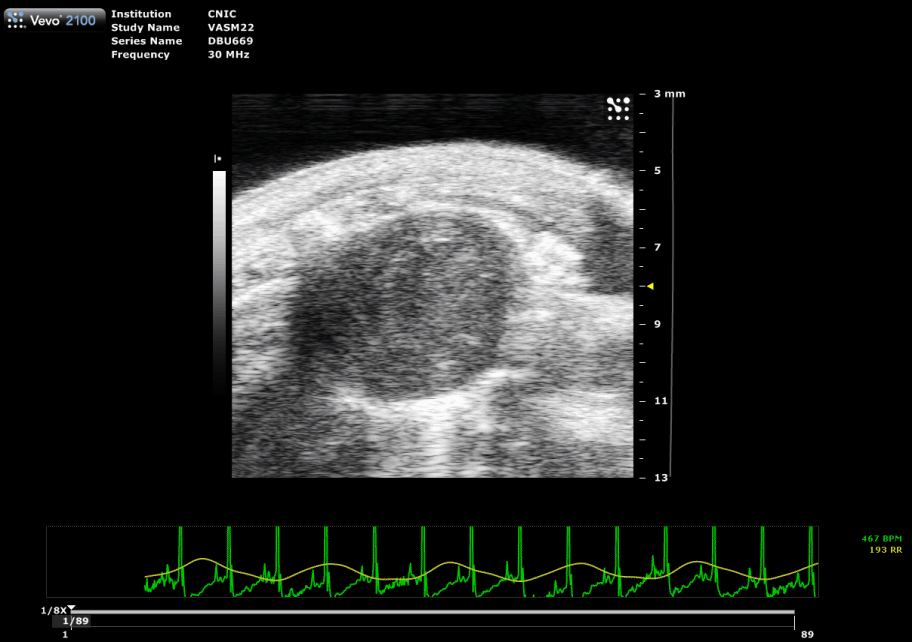

Supplement: Supplementary file 1 [file ijms-24-11172-s001.zip › Suppl video S1.gif]

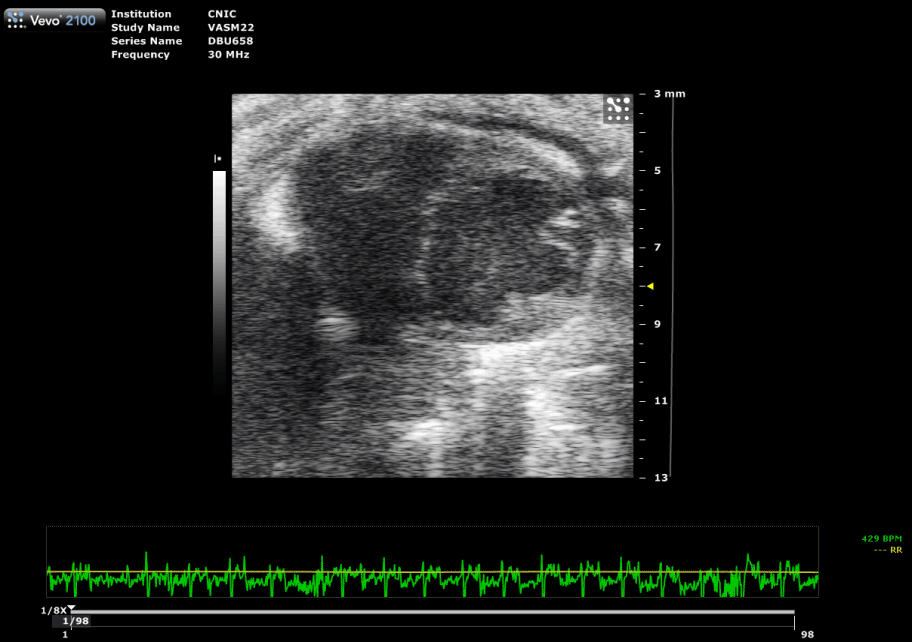

Supplement: Supplementary file 1 [file ijms-24-11172-s001.zip › Suppl video S2.gif]

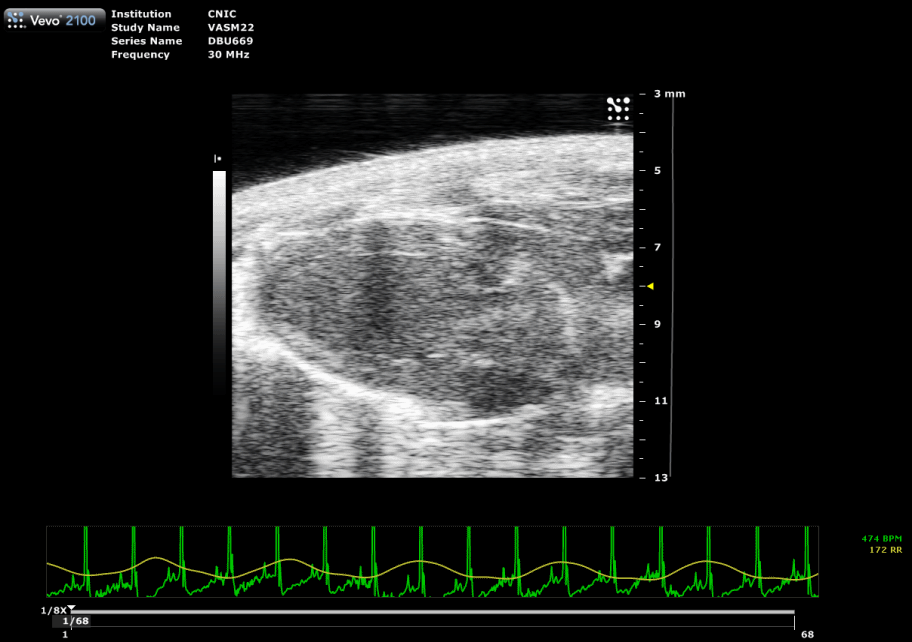

Supplement: Supplementary file 1 [file ijms-24-11172-s001.zip › Suppl video S3.gif]

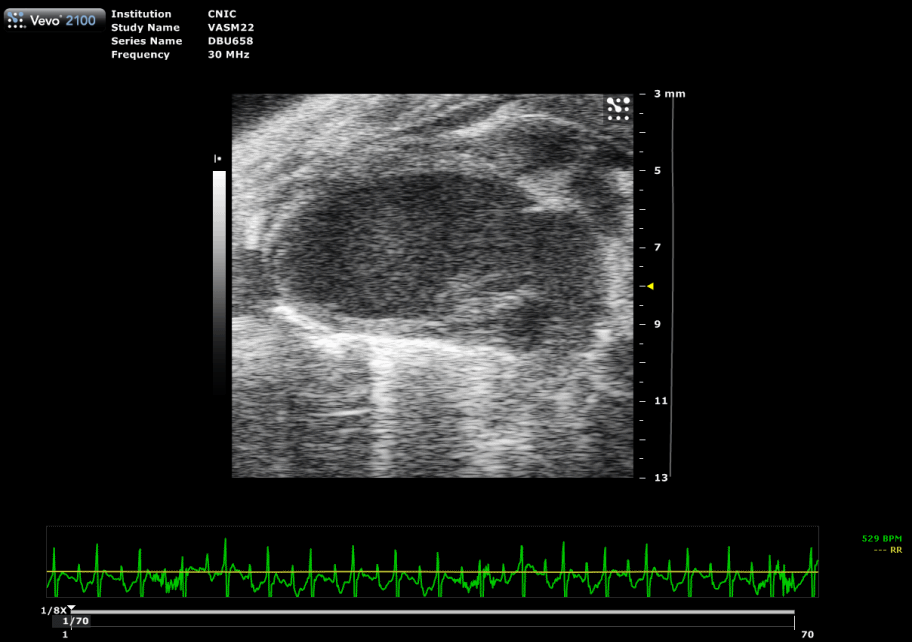

Supplement: Supplementary file 1 [file ijms-24-11172-s001.zip › Suppl video S4.gif]
